# CO-CREATING EVIDENCE-BASED PROGRAMMING AND EDUCATIONAL MATERIALS FOR ALASKA NATIVE CAREGIVERS

**DOI:** 10.1093/geroni/igae098.2016

**Published:** 2024-12-31

**Authors:** Steffi Kim, Jordan Lewis, Lena Thompson

**Affiliations:** University of Alaska, Anchorage, Alaska, United States; University of Alaska Fairbanks, College of Indigenous Studies, Fairbanks, Alaska, United States; University of Minnesota Duluth, Duluth, Minnesota, United States

## Abstract

Alaska Native Elders are rarely involved in creating educational and outreach materials. Over and over, Elders share that available educational and informative materials do not include their cultural understandings, programs do not meet their needs, and resource developers rarely attend to incorporate the specific and unique Alaska Native context. We will share our expereinces about outreach, recruitment, and establishment of an Alaska Native Community-engaged Aging Collaborative to engage community members, caregivers, and local organizations to create culturally appropriate and context-specific educational materials and programming that support Alaska Native Elders. Establishing this collaboration is the foundation for implementing research and programs driven by community needs and cultural understandings in conjunction with Western evidence-based approaches. Decolonizing strategies such as community member involvement, building trust, and sustainable relationships are not just steps but integral components of conducting Indigenous-driven research that benefits communities. These approaches not only sustain Elder participation in research related to aging well and dementia but also underscore the value and importance of their contributions. Indigenous research paradigms are gaining traction and hold immense potential in involving Indigenous older adults in research endeavors. Building these collaborations not only gives Elders a voice but also offers researchers a promising direction and invaluable insight into the specific needs of these communities, fostering a sense of hope and optimism for the future of research.

